# Repeatable aversion across threat types is linked with life-history traits but is dependent on how aversion is measured

**DOI:** 10.1098/rsos.172218

**Published:** 2018-02-28

**Authors:** Gabrielle L. Davidson, Michael S. Reichert, Jodie M. S. Crane, William O'Shea, John L. Quinn

**Affiliations:** Biological, Earth and Environmental Sciences, University College Cork, Cork, Ireland

**Keywords:** personality, gaze aversion, risk-taking, great tits, body condition, life history

## Abstract

Personality research suggests that individual differences in risk aversion may be explained by links with life-history variation. However, few empirical studies examine whether repeatable differences in risk avoidance behaviour covary with life-history traits among individuals in natural populations, or how these links vary depending on the context and the way risk aversion is measured. We measured two different risk avoidance behaviours (latency to enter the nest and inspection time) in wild great tits (*Parus major*) in two different contexts—response to a novel object and to a predator cue placed at the nest-box during incubation---and related these behaviours to female reproductive success and condition. Females responded equally strongly to both stimuli, and although both behaviours were repeatable, they did not correlate. Latency to enter was negatively related to body condition and the number of offspring fledged. By contrast, inspection time was directly explained by whether incubating females had been flushed from the nest before the trial began. Thus, our inferences on the relationship between risk aversion and fitness depend on how risk aversion was measured. Our results highlight the limitations of drawing conclusions about the relevance of single measures of a personality trait such as risk aversion.

## 1. Introduction

Assessing risk accurately and responding appropriately can optimize trade-offs between allocating resources to risk management and to other functional behaviours [1]. How individuals behave in this context is influenced by different mechanisms, including motivation to access food or invest in raising young (e.g. [2]), cognitive processes such as learning and categorization of threats (e.g. [3,4]) and constraints caused by correlations with other behavioural traits, as invoked by the ideas of personality and behavioural syndromes (e.g. [5]). Predation risk theory predicts that selection should favour animals that are plastic in their response to risk such that each individual responds at the same optimal level across contexts [1], while animal personality research has demonstrated that in reality individuals differ consistently in their risk-taking responses across time and contexts [6,7]. Several authors have suggested adaptive explanations for why individuals do not always behave optimally, in particular that individual differences are maintained in populations due to links with life-history variation (e.g. [7–9]). Empirical studies that link anti-predator behaviour, neophobia (i.e. the fear of novelty), life-history variation and fitness in natural populations are scarce (e.g. [10,11]), but essential to understanding how individual variation in risk aversion is maintained [12,13].

Individual variation in response to environmental stimuli has been reported in a variety of taxa, and this variation is consistent temporally, and across different contexts including responses to novel environments, to novel objects, to human disturbance and to predators (reviewed in [6,7]). Individuals that explore novel rooms more readily are often quicker to approach novel objects (e.g. [14–16]), and less perturbed by predation-like events [17–19]. As a result, terminology such as ‘bold’ and ‘shy’ has been used to broadly define a continuous behavioural axis, with high risk-taking individuals towards one end, and risk-averse individuals towards the other. These correlations between different traits that are hypothesized to characterize a behavioural axis are not universal, perhaps for adaptive reasons (e.g. [20]), but also because the traits involved have not always been clearly characterized (e.g. [21]). For example, jackdaws (*Corvus monedula*) that were more exploratory had higher object neophobia [22], but this may have been because high levels of movement around a novel room was an indication of anxiety, rather than exploratory boldness [4]. Other studies show that aversion towards novelty and to predator cues are not necessarily representative of the same personality axis (e.g. [23,24]). In rooks (*Corvus frugilegus*), neophobic responses towards novel objects were consistent within seasons, but not between seasons; whereas responses towards unfamiliar humans were consistent both within and between seasons [25]. To accurately characterize a behaviour as part of the risk-taking personality axis, it is important to consider, first, whether the behavioural response measured has been validated and properly categorized, second, the context (e.g. season or environment) and, third, how animals perceive the cues presented (i.e. do they classify them as novel or as predators) (reviewed in [4] and [26]).

Typically, individuals adjust their risk-taking behaviour in response to a wide variety of factors, including their own experience and the type of threat (e.g. [1,27]). Attending to direct predatory cues can alert animals to an impending attack, and several studies have shown that prey species respond aversely to the presence of eyes ([28,29] and references therein). Although conspecifics and many heterospecifics may also represent a threat, the most common interpretation is that eyes are a stimulus that elicits fearful responses because they represent a predator (reviewed in [28]; but see [30]). Animals show the strongest anti-predator responses to eyes directed towards them, rather than away [31,32], to eyes that are made to appear larger [33] and to configurations and orientations that most resemble eyes, as opposed to conspicuous shapes [34–36]. It has also been suggested that animals can learn to recognize eyes as predatory cues [29] because many species adjust their responses based on previous experiences and current information [3,37–40]. Even if individuals behave optimally through behavioural flexibility, rank order differences between individuals may still occur across risk-gradients.

Numerous mechanisms have been suggested to explain variation in risk aversion within populations, including the idea that personality reflects individual variation in life histories and/or current state [8,41]. One theory suggests that personality can be explained by individual variation in pace of life, for example in terms of how individuals trade off current and future reproductive success [42]. Support for this theory comes from theoretical models (e.g. [8,43]) as well as empirical evidence (e.g. [11,44,45]). Although not necessarily mutually exclusive from pace of life theory, state-dependent theory suggests that individual differences in traits arise when morphological or physiological differences determine how individuals behave [5,9]. Thus resource availability can have a direct influence on an individual's state and/or their perception of favourable versus unfavourable conditions. As a result, environmental factors can also mediate individual differences in behaviours such as risk-taking if some individuals are more sensitive to environmental change than others (e.g. [46,47]). Although several studies have found links between life history and personality traits (reviewed in [42,48]), others have found opposing relationships (e.g. [49]) or none at all, or depend on the personality trait measured (e.g. [50]). Studies that investigate the causes and consequences of personality trait differences in natural populations are an important step in understanding why personality traits arise, yet are limited (see [51] for a meta-analysis), and rarely investigate the links between personality traits like risk aversion, body condition and reproductive success in wild populations (but see [50,52,53]).

Great tits (*Parus major*) are an ideal species to investigate the relationship between threat-classification, personality and life-history variation. Great tits have shown consistent individual differences in boldness towards novel objects in the wild [16,52] and in captivity [14,15], and respond aversively to predator-like stimuli including eyes [36]. Our aim in this study was to determine the generality of risk aversiveness as a personality trait in great tits. To test this, we exposed females sequentially to a novel object and predatory cue (an image of sparrowhawk, *Accipiter nisus,* eyes) at the nest-box during the breeding season, and measured two different behavioural responses when females returned to the nest to incubate. First, we tested whether females classify the two stimulus types as distinct (i.e. behaved differently towards them). Second, we tested whether there was consistent between-individual variation in response across the two stimulus types that would indicate a repeatable, generalized ‘risk-taking’ personality trait across novelty and predatory cues. Moreover, if the two behaviours we measured covaried, this would indicate they are both components of the same personality trait. Finally, we investigated whether individual nest defence behaviour was associated with female body condition and reproductive output as potential causes or consequences of individual variation in personality.

## 2. Material and methods

### 2.1. Study site and nest-boxes

Our study took place across nine sites in the Bandon Valley, Co. Cork, Ireland, each separated by a distance of at least 2 km. Seven sites were considered mixed deciduous and two were conifer plantations. Across these sites, 286 nest-boxes suitable for great tits were distributed, hung at approximately 1.5 m above the ground and approximately 50 m apart (O'Shea *et al*. unpublished). Nest-boxes were monitored during the breeding season (April–June 2016) to determine egg lay-dates, clutch size, brood size and fledging success. Adults were caught at the nest at day 10 post-hatching to tag individuals and collect biometrics, including wing length and body weight to calculate body condition. Experiments took place between day 8 and 10 of incubation (where day 1 was the day following the date the last egg was laid). Permission to conduct fieldwork was provided by the National Parks and Wildlife Service (010/2016 and 004/2016) and Coillte Forestry.

### 2.2. Experimental procedure

#### 2.2.1. Experimental treatments

**Figure 1.**
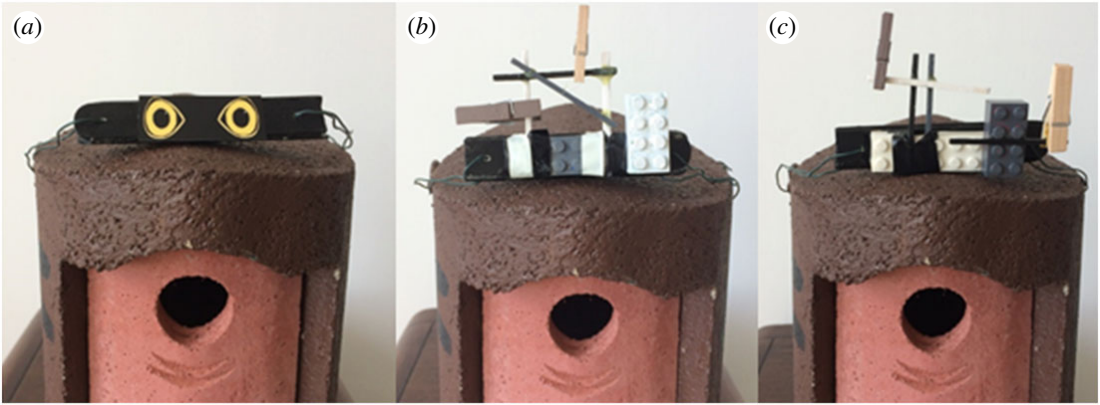
(*a*) The predator eyes and (*b,c*) novel object displayed on the nest-box.

Aversive stimuli were placed on the roof of 38 nest-boxes. All experimental boxes received two different treatments on consecutive days: a novel object and predator eyes. A subset of the same boxes (*n* = 22) received a third treatment of either (i) a repeat of a similar novel object (*n* = 12) to test whether their responses were repeatable and therefore an indication of a consistent personality trait, or (ii) nothing on the nest-box as a control, to test whether birds responded to disturbance from the experimental set-up (see below for details). Four additional boxes received only a control condition to increase sample size (*n* = 14). Therefore a total of 42 nest-boxes received at least one experimental treatment, and order of treatments was randomized. Not all boxes received a third treatment to minimize disturbance at the nest, which was also the reason we chose not to give repeat presentations of the predator cue. The predation cue was generated from a photograph of a sparrowhawk, the most important predator of the great tit [54], that was edited to isolate the eyes and remove shading artefacts using GNU Image Manipulation Program software, and printed onto waterproof Zecom® paper (figure 1*a*). Great tits have shown anti-predator responses to eye shapes on moth wings, and these aversive responses are stronger than equivalently conspicuous patterns, suggesting the potential for great tits to perceive eyes as predators [36]. The two novel objects consisted of different configurations of Lego® pieces, clothes pins and match sticks painted black and white, and covered with white and black electrical tape (figure 1*b,c*). The objects and the eyes were of similar size and mounted onto flat, black-painted wooden sticks (9 × 15 cm), and attached to the roof of the nest-boxes with garden wire.

Once the stimulus (or control) was secured to the nest-box, the experimenter removed the nest-box front (i.e. face plate) to determine if the female was present, and allowed the female to leave the box. If she did not leave the box within 20 s, a small wooden dowel was used to lift the tail gently. If the female still did not leave the nest, the face plate was returned and the experiment was not performed at that box. Therefore, all trials started with the female outside of the nest-box. In 33 of 96 trials, the female was not incubating when the experimenter arrived (10 eyes, 18 object, 5 control), and in 20 of these 33 trials at least one parent was alarm calling in proximity while the experiment was set up (7 eyes, 10 object, 2 control). If the female was incubating when the experimenter opened the face place, we recorded that she had been flushed from the nest by the experimenter as we expected that being startled could have a bearing on subsequent responses. Once the bird had left the box or it was confirmed that there was no bird inside, the experimenter replaced the face plate and left the area. All trials were recorded using a Panasonic HC-V250EB-K camera mounted on a tripod, covered in camouflage tape and positioned behind foliage 10 m from the nest-box. The trial ended after 40 min at which point the stimulus was removed from the nest-box, and the experimenter confirmed whether the female had returned by opening the face plate just enough to see inside, without causing her to flush. Trials occurred between 08.00 and 18.00, except for one box tested at 19.45. Trials at the same nest-box on different days were typically performed at a similar time of day (within a 2 h time frame). Four trials (one eyes, three object) were excluded from the analysis due to camera failure, and two boxes did not receive their last trial due to stoat (*Mustela erminea*) predation that occurred outside of the experimental treatment. The final dataset had 96 trials across 42 nest-boxes. Ten nests failed at a later date after the conclusion of the experiment, five were due to predation, and the rest of unknown causes. All other nests were successful. The rate of nest failures was similar to those recorded in areas not receiving experimental trials (i.e. 12 of 46 nests).

#### 2.2.2. Behavioural analysis

We recorded behaviours for females only because male great tits do not incubate [54]. Males range widely over their territory during incubation and return primarily to perch outside the nest cavity to feed the female [54]. The following two behaviours were analysed from the video footage: (i) latency to enter the nest-box, starting from the time the nest-box front was placed back on the box, and (ii) inspection time, the total duration spent perched at the nest entrance hole looking inside and outside of the box before deciding to enter or fly away. Females were identified by plumage characteristics, and in cases where this was not visible, by their behaviour (i.e. birds were confirmed to be female if they remained inside the nest for 10 min following entry as an indication that they were incubating). There were two instances when males came to the box before females, and in both cases their behaviour differed to that of females in that they carried food in their beak and made calls for the absent female. Twenty per cent of videos were analysed by a second coder. Inter-rater reliability was assessed using a two-way intraclass correlation coefficient (ICC) for agreement; latency to enter ICC= 1.0, *p *< 0.001; inspection time ICC = 0.99, *p *< 0.001.

### 2.3. Statistical analysis

#### 2.3.1. Behavioural responses to stimuli

We tested whether the two behavioural responses differed across the three treatments at the nest-box. Only birds that landed at the nest entrance or approached the nest-box were included in the analysis for inspection time (total trials = 75). We ran separate analyses with each of the behaviours as the response variable. For our latency to enter variable (total trials = 96), birds that did not enter the nest during the trial period were given an upper latency of 2400 s (i.e. 40 min). Data were log-transformed and analysed using generalized linear mixed models (GLMMs) fitted to a Gaussian distribution with a log link function. The following fixed effects were included in our model as potential influences on aversion behaviours: treatment (control, eyes, object); flush: Y or N (i.e. whether the female was incubating and flew out of the nest during the experimental set-up); clutch size (because her current investment may affect her motivation to return to incubate, or as a potential link to a life-history trait); presentation order (as birds may reduce responses to experimental treatment across successive trials); lay date (as females may perceive their reproductive potential as greater earlier in the season which may influence risk aversion); time of day; and treatment × flush interaction. Female ID and woodland site were included as random factors. We present the main effects from the full models and only include interactions when significant. Alpha was set at 0.05 and terms with a *p*-value less than 0.10 are discussed as trends. The control stimulus was set as the reference category, and post hoc Tukey's tests were performed if our treatment variable was significant, to test for differences between eyes and object, and to account for multiple comparisons. All GLMMs were run in the lme4 package [55] for R statistical software [56] after checking that they met model assumptions (homogeneity and normality of residuals), while post hoc comparisons were run using the multicomp package [57].

To determine individual repeatability for each of the two behavioural responses, we calculated repeatability following [58] by dividing the individual variance by the total variance extracted from the GLMM. In addition, we also report adjusted repeatability, as this method controls for factors that may influence individual behaviour by including fixed effects that came out as significant from our first analyses on behavioural responses to stimuli. For both adjusted and non-adjusted repeatability, individual ID and woodland site were included as random effects. Significance for repeatability was obtained by performing a log-likelihood ratio test between the model as described above and the same model with ID excluded as a random term.

To test whether latency to enter and inspection time correlated, and whether these behavioural measures were linked with fitness and body condition, we extracted a single value for each individual for each of our behavioural measures. Following methods used in [59], we ran a GLMM with fixed factors that were significant in our analyses described above as well as female ID in order to obtain parameter estimates for each individual. The parameter estimate for each individual was added to the model constant (i.e. intercept) which gives an estimate with respect to the fixed effects. An alternative method would be to run a multivariate analysis with our behavioural and fitness measures as response variables, and our fixed effects as our explanatory variables. However, given our samples size, our dataset does not have the power necessary for such analyses [60]. Thus, we note that the results obtained using our estimates for aversion behaviour should be treated with caution given that extracted individual values from model estimates can lead to bias and anticonservatism [61,62].

Using our estimates for aversion behaviour, we tested whether the two behavioural measures (latency to enter and inspection time) were correlated using a GLMM as described above, with site as a random term.

#### 2.3.2. Links between aversion behaviour, reproductive success and body condition

We predicted that if levels of aversion to the stimuli were linked to fitness, then our individual estimates for latency to enter the nest and/or inspection time at the nest should be linked to the number of fledglings. The total number of fledged chicks was analysed using a GLMM fitted to a Gaussian distribution with a log link function. Lay date was included as a fixed factor to control for any effects of timing of breeding on reproductive success [53,63], and site was included as a random factor. We did not include age in our analysis because all individuals were adults, aside from two juveniles. Because nine nests failed before adults could be measured, we excluded female body condition as a potential influence on reproductive success to increase sample size. Moreover, a separate analysis showed that body condition had no significant effect on fledgling success in our population, both when we used a restricted dataset excluding these nine birds as well as when we used an expanded dataset (*n* = 56) including birds that we had biometrics for, even if they had not received experimental treatments at the nest-box.

We ran a separate analysis on the nests where we had data to infer female body condition to determine whether body condition was linked to our individual estimates for latency to enter and inspection time. We predicted that females may be more risk averse if they were in poorer body condition. We determined female body condition using the scale mass index following Peig & Green [64], which standardizes body mass at a fixed value of tarsus length, and is shown to be a better predictor of relative size and energy reserves than other conventional methods including ordinary least squares residuals [64,65]. We scaled the females from our experimental boxes against all females measured in our field sites to obtain the most accurate average value for tarsus and body weight from the study population. Data were analysed using a GLMM fitted to a Gaussian distribution with a log link function. Body condition and lay date were included as fixed effects and site as a random term.

## 3. Results

### 3.1. Behavioural responses

**Figure 2.**
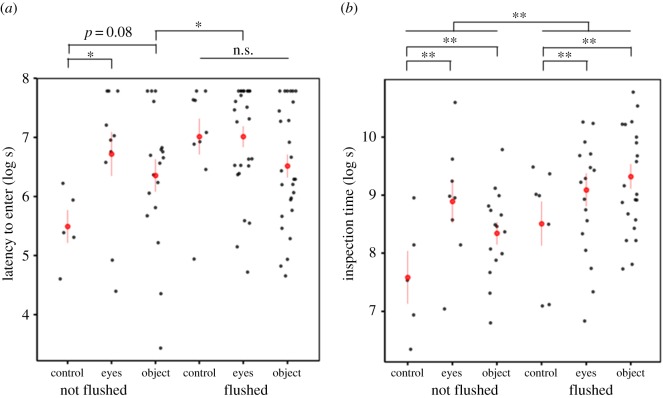
(*a*) Latency to enter the nest-box; (*b*) total inspection time once landed on the nest-box. Red dot and lines denotes mean ± s.e. from log-transformed data. *p < 0.05, **p < 0.01. Two outlier points were removed from graph for scale purposes in (*b*) (eyes, flushed 150 s; object, flushed 86 s).

Latency to enter was influenced by an interaction between stimulus treatments and whether birds were flushed (eyes × flush, object × flush table 1*a* and figure 2). In the control conditions, birds quickly entered the nest when not flushed, but flushed birds took longer to enter the nest. Latency to enter did not differ between control birds that were flushed and the eye and object treatments (whether or not the birds were flushed in these two treatments). However, birds in the eye treatment that were flushed took longer to enter than birds in the object treatment that were not flushed. There were no other differences in latency to enter for birds in the eyes and object treatments, whether or not they had been flushed from the nest (table 2 for post hoc results). Birds that laid earlier in the season were quicker to enter the nest (lay date table 1*a*). There was no effect of clutch size, presentation order or time of day (table 1*a*).

**Table 1.** Full model outputs from GLMMs with factors affecting, at the population level, (*a*) latency to enter and (*b*) inspection time. At the individual level, factors affecting fledgling number (individual estimates for latency to enter (*c*) and inspection time (*d*)), and the effect of body condition on individual estimates for latency to enter (*e*), and inspection time (*f*). Please refer to Material and methods for details of factor inclusion for each model. Full models are presented with interactions included only when significant.

model	subjects	d.f.	fixed and random effects	*β* ± s.e.	*t*	*p*	variance (adjusted, non-adjusted)
(a) latency to enter	96	42	intercept	2.55 ± 1.41	1.81	0.08	
			eyes	1.41 ± 0.43	3.32	<0.01	
			object	1.03 ± 0.39	2.66	0.01	
			flush	1.53 ± 0.45	3.42	<0.01	
			treatment × eyes	−1.13 ± 0.54	−2.10	0.04	
			treatment × object	−1.16 ± 0.50	−2.31	0.03	
			lay date	0.05 ± 0.02	2.83	<0.01	
			clutch size	−0.18 ± 0.11	−1.60	0.12	
			presentation order	−0.06 ± 0.10	−0.63	0.53	
			time of day	0.05 ± 0.04	1.25	0.22	
			box				0.23,0.27
			site				0.23,0.27
			residual				0.44,0.53
(b) inspection time	75	36	intercept	7.10 ± 1.76	4.02	<0.001	
			eyes	1.03 ± 0.34	3.06	<0.01	
			object	0.98 ± 0.32	3.06	<0.01	
			flush	0.78 ± 0.23	3.46	<0.01	
			presentation order	−0.39 ± 0.13	−3.10	<0.01	
			time of day	0.08 ± 0.05	1.51	0.14	
			lay date	0.00 ± 0.02	0.10	0.92	
			clutch size	0.01 ± 0.12	0.05	0.96	
			box				0.14,0.27
			site				0.14,0.27
			residual				0.67,0.99
(c) fledgling no.	38	28	intercept	4.99 ± 2.86	1.75	0.09	
			latency to enter	−0.77 ± 0.39	−1.96	0.06	
			lay date	0.03 ± 0.04	0.67	0.51	
(d) fledgling no.	31	21	intercept	4.84 ± 4.53	1.07	0.30	
			inspection time	−0.31 ± 0.38	−0.82	0.42	
			lay date	0.02 ± 0.05	0.31	0.76	
(e) latency to enter	30	21	intercept	9.42 ± 3.67	2.57	0.02	
			body condition	−0.38 ± 0.16	−2.38	0.03	
			lay date	0.03 ± 0.02	1.47	0.16	
(f) inspection time	26	17	intercept	10.52 ± 3.94	2.67	0.02	
			body condition	−0.09 ± 0.17	−0.55	0.60	
			lay date	−0.01 ± 0.02	−0.51	0.62

**Table 2.** Post hoc Tukey's tests for latency to enter the nest-box between object, eyes and control and trials in which the female was (Y) or was not (N) flushed from the box.

post hoc Tukey's tests	eyes (Y)	eyes (N)	object (Y)	object (N)	control (Y)
eyes (N)	*z* = 1.34, *p* = 0.75				
object (Y)	*z *= −2.03, *p* = 0.31	*z *< −0.01, *p* = 0.99			
object (N)	*z* = 3.28, *p* < 0.05	*z *= −1.29, *p* = 0.78	*z* = 1.53, *p* = 0.62		
control (Y)	*z* = 0.90, *p* = 0.94	*z* = 0.33, *p* = 0.99	*z*=−0.39, *p* = 0.99	*z* = 1.46, *p* = 0.67	
control (N)	*z* = 4.84, *p* < 0.001	*z* = 3.32, *p* = 0.01	*z* = 3.71, *p* <0.01	*z* = 2.66, *p* = 0.08	*z* = 3.41, *p* < 0.01

Inspection time was higher in the eyes and object treatments compared to the control (table 1*b* and figure 2*b*). There was no difference in inspection time between eyes and object (post hoc Tukey's test: *z* = −0.24, *p* = 0.97). When females were flushed, inspection times were greater across all treatments and inspection times decreased significantly after successive presentations (table 1*b*). Similar results for all fixed factors were obtained when two visually obvious outliers (figure 2*b*) were removed.

We did not find a correlation between latency to enter and inspection time suggesting that these two behaviours do not form part of the same personality axis (*β* ± s.e. = 0.03 ± 0.17, *z* = 0.16, *p* = 0.88). Similar results were obtained when the two visually obvious outliers for inspection time were removed, though we had no *a priori* reason to remove those outliers.

### 3.2. Individual repeatability

Latency to enter the nest-box was significantly repeatable among individuals for object and eye treatments, when controlling for lay date and the stimulus by flush interaction (adjusted repeatability *R* = 0.25, *p *< 0.01, CI = 0.33, 0.85), and when not controlling for these factors (non-adjusted repeatability *R* = 0.25, *p* = 0.03, CI = 0.31, 0.82). Individual repeatability for inspection time was marginally significant (*R* = 0.14, *p* = 0.05, CI = 0.0, 0.84) while controlling for flush and presentation order, but this effect was not significant when two visually obvious outliers were removed (*R* = 0.11, *p* = 0.11, CI = 0.0, 0.74), and when not controlling for significant fixed factors (non-adjusted repeatability: *R* = 0.07, *p* = 0.39, CI = 0.0, 0.74).

### 3.3. Fitness measures and latency to enter

**Figure 3.**
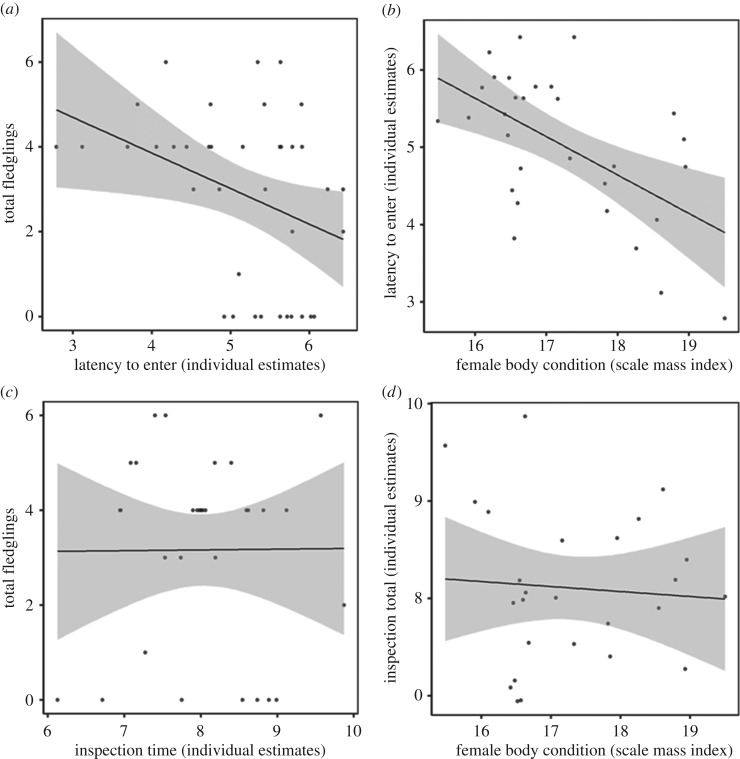
(*a*) A non-significant trend for a relationship between latency to enter and number of fledglings. (*b*) Latency to enter significantly predicts female body condition. Inspection time does not correlate with number of fledglings (*c*) or body condition (*d*). Shaded area denotes 95% confidence interval. Data for latency to enter and inspection time are estimates extracted from a GLMM to control for fixed effects whereby larger values reflect longer latency and inspection times.

Females that had lower latency to enter tended to have more fledglings than those that had higher latency to enter (table 1*c* and figure 3*a*). Fledgling number was not influenced by lay date. Females in poorer body condition had higher latency to enter than ones in better body condition (figure 3*b* and table 1*e*). Body condition was not influenced by lay date. Inspection time was not related to fledgling number or body condition (figure 3*c,d* and table 1*d,f*).

## 4. Discussion

This study addressed three questions regarding animal responses to threatening stimuli. First, we asked whether a predator cue elicits stronger responses than a novel object (or vice versa), second, whether individuals expressed consistent and repeatable behaviour across stimulus types and third, whether this behaviour was linked to individual state and fitness-related traits. Great tits responded similarly to a predator cue and to a novel object, but latency to enter was not related to our other response measure, inspection time. Moreover, repeatability was higher for latency to enter than inspection time. Latency to enter the nest-box was negatively related to body condition, but was unrelated to clutch size or fledgling success, although a non-significant trend suggests that risk-prone females may have produced more fledglings. By contrast, there were no links between inspection time and our fitness measures.

### 4.1. Risk aversion, context and consistency

Many animals respond flexibly to threats because they have learned through experience and/or they classify specific features into categories such as novel, predatory or non-threatening (e.g. [4,38,40]). Our results suggest that great tits generalize their fear responses to both novel and predator stimuli. This may be because they are constrained within their personality type to show the same level of aversion regardless of the threat. This is supported by the fact that for most treatments, latencies to enter the box were similar whether or not they had experienced being flushed from the nest by the experimenter. However, females were quicker to come back to the nest when the object was on the box and they had not experienced being flushed compared to when the eyes were on the box and they had experienced being flushed, perhaps because there was a synergistic effect of treatment type and human disturbance at the box. Hormonal response related to stress is a potential mechanism that may mediate how long it takes a female to overcome fear and return to the nest-box. Peak corticosterone (CORT) concentrations in the bloodstream following an acute stressor have been shown to correlate with boldness and neophobia [66,67]. However, because blood sampling involves predator-like handling from the experimenter, it is unknown whether a similar concentration of CORT is released into the blood stream in response to non-predator threats such as novelty [53].

Latencies to enter the nest-box and inspection times may also be similar across eye and novel object treatments because individuals have not had enough experience with, or have not adapted to high frequencies of encounters with either novel objects or predators. Rural birds have been reported to be more neophobic than urban birds [68,69], which may explain why birds in our rural population treated a novel object as equally threatening as the predator stimulus. An alternative, perhaps more intuitive explanation is that great tits classified both stimuli as novel. Although we modelled our eye stimulus from a natural great tit predator, the eyes may have been perceived as novel because they were isolated and printed to paper, or alternatively, an unwanted object on the nest-box (regardless of appearance), was treated as a novel situation/changed environment. The perception mechanisms underlying gaze aversion to eye shapes are still under debate, namely whether eyes represent predators, or whether they elicit alarm responses simply because they are conspicuous (reviewed in [26,30]). Nevertheless, when tested in captivity in a foraging context, birds showed the strongest alarm responses to the sudden appearance of two-dimensional images of a known predator and to isolated eye-spots from the same predator, but not to conspicuous, non-predator shapes [36]. Although eyes may be perceived as a potential predator cue when first detected by prey, it is unknown whether this remains the case following further inspection. Repeated presentations of different predator cues and predator models would be necessary to validate whether aversion behaviours in our study were due to the stimulus being perceived as novel, predatory or just generally risky. These alternative interpretations regarding classification and perception of threat reiterate the difficulty in disentangling the underlying mechanisms that shape aversion behaviours [4].

Previous studies measure risk aversion using a variety of different traits, including latency to enter the nest-box (e.g. [3,16,53]) and inspection time (e.g. [23,70]). Our results show that very different conclusions can be drawn depending on which of these two variables are used. Inspection time was significantly greater if birds had been flushed from the nest regardless of the presence of, or type of stimulus on their box, demonstrating that this behaviour is linked to the experience of an acute threat. By contrast, latency to enter was robust to most experimental treatments and variables, and more of the phenotypic variation was explained by differences among individuals compared to inspection time (*R* = 0.25 compared to *R* = 0.14 or *R* = 0.07 unadjusted). Moreover, latency to enter and inspection time did not correlate, suggesting they do not form part of the same behavioural syndrome. Therefore inspection time may not be a measure of risk aversion in the context of the bold–shy personality axis. Instead, inspection time may have measured females' decision-making time while they assessed the safety and contents of the nest, a behaviour that was sensitive to a number of extrinsic factors. Inspection time increased if birds were flushed from the nest, and decreased over successive presentations, indicating that this behaviour is plastic depending on the context. The lack of covariation between behaviours thought to measure risk-taking has previously been reported in great tits, suggesting that domain-specific as opposed to domain-general processes may operate in order to serve different functions when assessing risk [71]. Risk-taking behaviours may involve different underlying mechanisms depending on the context, but whether these are physiological, cognitive or otherwise is uncertain. As a whole, these results illustrate the importance of validating multiple behavioural measures when trying to ascertain whether particular aversion behaviours are components of the same personality trait (see [26,72] for similar arguments).

### 4.2. Fitness, current state and mechanisms

How individuals behave when they perceive a threat may determine whether they avoid predation, or gain access to resources, two outcomes that have direct links with fitness (e.g. [73]). Here we showed that in the context of latency to enter the nest-box, risk-prone females were in better body condition and risk-prone individuals tended to have more fledglings than risk-averse females (independent of their body condition). However, these results should be treated with caution because they were obtained using model estimates of individual aversion that have the potential to lead to type I errors [61,62]. Nevertheless, we discuss what our results mean for our understanding of the significance of risk aversion in the context of personality theory. The pace of life theory of personality states that differences in risk aversion arise because of alternative life-history strategies, where risk-prone individuals prioritize current reproductive investment at the potential cost of future investment, while those individuals at the risk-averse end of the continuum do the opposite (e.g. [8,42,48]). Many empirical studies have focused on these trade-offs in a wide variety of contexts including fecundity, growth and starvation (e.g. [11,74–76]). In this study, although individuals that were quicker to enter the nest did not produce larger clutches as predicted by the pace of life theory, they did lay earlier and tended to produce more fledglings. However, there was no evidence they did so at the cost of their own viability, and on the contrary risk-prone individuals were in better condition. Therefore, the causal relationship could have been that body condition determined risk-taking behaviour and subsequent reproductive investment [5,9], in line with state-dependent theory [5] and traditional optimal life-history theory [77–79]. Although not measured in this study, physiological traits such as hormonal responses and metabolic rate would be informative as to whether aversion behaviour was linked to life-history variation. Another explanation for links between fitness traits and risk-taking is that risk-taking behaviour is linked to individual sensitivity to environmental cues (e.g. [46,47]) and that risk-averse individuals may have been more sensitive to reduced foraging opportunities or perceived reproductive potential. Indeed, on average females took longer to return to the nest later in the season, suggesting they may have been more risk averse in response to declining resource availability. Similarly, a link with body condition due to resource access could have been caused by covariation between risk and competitive ability [80]. However, we found no evidence that females were sensitive to their reproductive potential based on the number of eggs in their nest, as latency to enter the nest was unrelated to clutch size.

Although our measure of repeatable aversion is limited within a single season only, latency to enter has previously been reported to be related to exploration behaviour in another population [16], and was found to be heritable [59]. However, although latency to enter, and to some extent inspection time may be repeatable, their heritability may be very low or negligible, and most of the observed differences in risk-taking between individuals may have been caused by phenotypic plasticity, arising for example because of temporary local conditions (reviewed in [81]) or because of permanent environment effects during earlier life (e.g. [82,83]). Disentangling whether individual differences arise by selection acting on additive genetic variation, or through phenotypic plasticity remains challenging [84]. Longitudinal and experimental studies will be required to rule out confounding factors such as time of year and local environmental conditions, and quantitative genetic analysis would be required to determine whether the repeatability shown in this study has an additive genetic basis with consequences for microevolutionary processes in the population. Moreover, our study cautions that the presence or lack of such relationships can be dependent on the behaviour measured.

## Data accessibility

Data can be accessed at the Dryad Digital Repository: (http://dx.doi.org/10.5061/dryad.rt83q) [85].
